# Wnt5a Promotes Cortical Neuron Survival by Inhibiting Cell-Cycle Activation

**DOI:** 10.3389/fncel.2017.00281

**Published:** 2017-09-29

**Authors:** Li Zhou, Di Chen, Xu-Ming Huang, Fei Long, Hua Cai, Wen-Xia Yao, Zhong-Cheng Chen, Zhi-Jian Liao, Zhe-Zhi Deng, Sha Tan, Yi-Long Shan, Wei Cai, Yu-Ge Wang, Ri-Hong Yang, Nan Jiang, Tao Peng, Ming-Fan Hong, Zheng-Qi Lu

**Affiliations:** ^1^Department of Neurology, The Third Affiliated Hospital of Sun Yat-sen University, Guangzhou, China; ^2^Department of Rehabilitation, The First Affiliated Hospital of Clinical Medicine of Guangdong Pharmaceutical University, Guangzhou, China; ^3^Laboratory of Viral Immunology, State Key Laboratory of Respiratory Disease, Guangzhou Institutes of Biomedicine and Health, Chinese Academy of Sciences, Sino-French Hoffmann Institute of Immunology, Guangzhou Medical University, Guangzhou, China; ^4^Department of Laboratory, Third Affiliated Hospital of Sun Yat-sen University, Guangzhou, China; ^5^Institute of Hematology, Guangzhou, China; ^6^Department of Pathology, Third Affiliated Hospital of Sun Yat-sen University, Guangzhou, China; ^7^Department of Hepatic Surgery, Third Affiliated Hospital of Sun Yat-sen University, Guangzhou, China; ^8^Department of Neurology, The First Affiliated Hospital of Clinical Medicine of Guangdong Pharmaceutical University, Guangzhou, China

**Keywords:** Wnt5a, β-Amyloid protein, Alzheimer’s disease, cortical neuron, apoptosis, cell-cycle activation, Cyclin D1

## Abstract

β-Amyloid protein (Aβ) is thought to cause neuronal loss in Alzheimer’s disease (AD). Aβ treatment promotes the re-activation of a mitotic cycle and induces rapid apoptotic death of neurons. However, the signaling pathways mediating cell-cycle activation during neuron apoptosis have not been determined. We find that Wnt5a acts as a mediator of cortical neuron survival, and Aβ_42_ promotes cortical neuron apoptosis by downregulating the expression of Wnt5a. Cell-cycle activation is mediated by the reduced inhibitory effect of Wnt5a in Aβ_42_ treated cortical neurons. Furthermore, Wnt5a signals through the non-canonical Wnt/Ca^2+^ pathway to suppress cyclin D1 expression and negatively regulate neuronal cell-cycle activation in a cell-autonomous manner. Together, aberrant downregulation of Wnt5a signaling is a crucial step during Aβ_42_ induced cortical neuron apoptosis and might contribute to AD-related neurodegeneration.

## Introduction

Alzheimer’s disease (AD) is a chronic neurodegenerative disease that induces by progressive neuron loss (Davies and Maloney, 1976; Whitehouse et al., 1982; Gómez-Isla et al., 1996). The key events of AD pathogenesis are the production and deposition of amyloid-beta peptides (Aβ; Hardy and Selkoe, 2002; Murphy and LeVine, 2010). Aβ induces DNA synthesis and promotes the re-entry of cell-cycle in cultured cortical neurons and in mouse models of AD (Kruman et al., 2004; Varvel et al., 2008). Aberrant cell-cycle activation is causally related to neuronal apoptosis and might be a root cause of several neurodegenerative disorders (Liu and Greene, 2001; Yang et al., 2001, 2003; Becker and Bonni, 2004; Herrup and Yang, 2007). A series of the proteins report to be involved in AD pathology is cyclin-dependent kinases (CDKs) and their regulators (such as CDK1, CDK4, CDK5, p16, Cyclin B1, Cyclin D1 and Cyclin E; Arendt et al., 1996; McShea et al., 1997; Vincent et al., 1997; Hoozemans et al., 2002; Shukla et al., 2012). Meanwhile, previous studies demonstrated that Aβ peptides will lead to re-enter various phases of the cell cycle in adult neurons. Aβ induces the cleavage of CDK5 regulatory subunit p35 to p25 (Patrick et al., 1999), promotes the expression of Cyclin D1 and B1 (Majd et al., 2008), increases the activity of CDK1, CDK4 and CDK5 (Milton, 2002; Biswas et al., 2007; Lopes et al., 2010). However, the signaling pathways mediating CDKs activation during Aβ induced neuron apoptosis have not been determined.

Recently, more and more evidence suggest a strong relationship between a dysregulation of the Wnt signaling pathway activity and AD (De Ferrari et al., 2014; Riise et al., 2015). Many components of Wnt signaling are altered in AD, such as Dkk1 and β-catenin. Dkk1, which inhibits Wnt signaling by binding to the LRPs and preventing Wnts from forming a complex with Fz and LRPs (Zorn, 2001), is increased in brains of both familial/early-onset AD and sporadic/late-onset AD patients (De Ferrari and Moon, 2006; Boonen et al., 2009). DKK1 is also upregulated in cultured cortical neurons treated with Aβ (Caricasole et al., 2004). β-catenin is reduced in brains of AD patients carrying presenilin-1-inherited mutations (Zhang et al., 1998) and in brains of late-onset AD patients (De Ferrari et al., 2007).

Moreover, Wnt pathway plays an important role in the regulation of cell cycle (Davidson and Niehrs, 2010). Wnt1 blocks the differentiation and enhance the proliferation of PC12 cells by activating cyclin D1 (Issack and Ziff, 1998), the Cyclin D1 is also a direct target gene of the Wnt/β-catenin/LEF-1 pathway through a LEF-1 binding site in the Cyclin D1 promoter (Shtutman et al., 1999), Wnt pathway promotes oral squamous-cell carcinoma cell proliferation by negatively control the expression of the human homolog of the *Drosophila headcase* (HECA) and reduces the interaction of HECA with CDK2, CDK9, Cyclin A and Cyclin K (Dowejko et al., 2012) and so on. Based on the importance of cell-cycle reactivation in Aβ induced neuronal injury and the essential role of Wnt pathway in activation of cell cycle, we speculate that Aβ may activate the cell-cycle pathway by Wnt family members.

The purpose of this study was to investigate the role of Wnt family members in Aβ_42_ induced cortical neuron damage. This study is the first to show Aβ_42_ induced non-canonical Wnt5a/Ca^2+^ pathway dysfunction and cell-cycle re-action in cortical neurons. Meaningfully, we find Wnt5a can protect Aβ_42_ treated cortical neurons by alleviating the expression of Cyclin D1. These observations illuminate that aberrant downregulation of Wnt5a signaling is a crucial pathological step that contributes to AD-related neurodegeneration.

## Materials and Methods

### Reagents

Amyloid β peptide (1–42; Human Aβ_42_; Abcam plc., #ab120301), Hoechst 33258 (Sigma-Aldrich, #B2883), recombinant mouse Wnt5a protein (Bio-Techne China Co. Ltd. R&D Systems #645-WN), Roscovitine (ROS; Selleck.cn, #S1153), KN62 (Selleck.cn, #S7422), Calmodulin Kinase IINtide, Myristoylated (Ntide; Merck Millipore, #208921) and Z-VAD-FMK (Selleck.cn, #S7023) were added to the media at the indicated concentrations and time points. For transient gene transfection, DIC5 cortical neurons were transfected using the calcium phosphate transfection method described previously (Kingston et al., 2003). Lipofectamine 3000 (Thermo Fisher Scientific Inc.) was used for transient gene or siRNA transfection of HEK293 cells.

The following primary antibodies were used: Anti-Wnt7a antibody (Abcam plc., #ab100792), Anti-Wnt5a antibody (Abcam plc., #ab72583), Anti-beta Tubulin antibody (Abcam plc., #ab6046), Anti-Cyclin D1 antibody (Abcam plc., #ab16663), Anti-E2F1 antibody (Abcam plc., #ab94888), phospho-Rb (Ser795) antibody (Cell Signaling Technology, Inc., #9301), Anti-β-Catenin antibody (Abcam plc., #ab32572), Anti-Histone H1 antibody (Abcam plc., #ab71594), Anti-phospho-CaMKII alpha (Thr286)/beta (Thr287; Abcam plc., #ab32678), Flag (Sigma-Aldrich, #F1804).

### Cell Culture

Primary cortical neurons of either sex were established from cortices dissected from newborn C57/BL6 mice (<24 h) as described previously (Sciarretta and Minichiello, 2010). Animals were purchased from the Experimental Animal Center of Sun Yat-sen University. Ethical approval was obtained from the Animal Ethics Committee of Sun Yat-sen University. Briefly, the whole cerebral cortex was isolated and cells were dissociated in a trypsin solution (2.5 mg/mL in Hank’s buffer salt solution) for 10 min at 37°C. The cortex cell suspension was centrifuged and re-suspended, seeded at a density of 1.5 × 10^6^ cells/ml in plates pre-coated with poly-d-lysine (Sigma, St. Louis, MO, USA), and grown in Neurobasal™-A medium containing 2% B27 supplement (Thermo Fisher Scientific Inc., Waltham, MA, USA), L-glutamine (0.25 mM, Invitrogen), GlutaMax-I (0.25 mM, Thermo Fisher Scientific Inc.), 1% penicillin (100 U/ml, Invitrogen), 1% streptomycin (100 μg/ml, Invitrogen) in a humidified incubator with 5% CO_2_ at 37°C. HEK293 cells were obtained from the American Type Culture Collection (ATCC; Manassas, VA, USA) and were cultured at 37°C in 5% CO_2_ in Dulbecco’s modified Eagle’s medium (DMEM; Invitrogen) supplemented with 1% penicillin (100 U/ml, Invitrogen), 1% streptomycin (100 μg/ml, Invitrogen), L-glutamine (292 μg/ml, Invitrogen) and 10% fetal bovine serum (FBS; hyClone Laboratories).

### Pathological Studies

Human cortical tissues obtained from surgical resection of patients with IV grade glioblastoma (glioblastoma adjacent normal cortical tissues) were fixed in 4% paraformaldehyde overnight at 4°C. Before sampling, routine processing, and paraffin embedding were performed. Written informed consent had been obtained from the family members of patients before the surgery. The human tissue study was approved by the Ethics Committee of the Third Affiliated Hospital of Sun Yat-sen University.

### Animals and Drug Treatment

Male mice (8–12 week-old from Experimental Animal Center of Sun Yat-sen University, Guangzhou, China), maintained at an ambient temperature of 22–24°C under a 12:12 h light:dark cycle, were used in this experiment. Animals were divided into three groups (*n* = 10 each group): (1) sham-operated plus physiological saline treatment; (2) Human Aβ_42_ (3 nmol/10 μl) i.c.v. (intracerebroventricularly) injection plus physiological saline treatment; and (3) Human Aβ_42_ (3 nmol/10 μl) plus recombinant mouse Wnt5a protein treatment (6 nmol/10 μl) i.c.v. injection. To obtain the aggregated form of Aβ_42_, the peptide solution was placed in an incubator at 37°C for 72 h. Seven days after injection, mice were perfused transcardially with 4% paraformaldehyde in phosphate buffered saline (PBS). The brains were post fixed for 24 h and were embedded in paraffin wax. Serial coronal sections (5 μm thickness) were cut from various sections of the brain.

### Surgery

All experimental procedures were carried out in accordance with the guidelines of the Animal Care and Use Committee of Sun Yat-sen University. The mice weighing 20–24 g were anesthetized with sodium pentobarbital (Sigma-Aldrich Co., at a dose of 70 mg/kg) and placed in a stereotaxic apparatus (Stoelting, USA). According to the atlas of Paxinos, Franklin, and Franklin (Paxinos and Franklin’s the Mouse Brain in Stereotaxic Coordinates, Fourth Edition), 8 mm 26-gauge stainless-steel guide cannulas, closed by stylets, were implanted over the lateral ventricle (0.5 mm posterior to bregma, 1.0 mm lateral to midline, 2.0 mm ventral to skull surface). Body temperature of the mice was maintained at 37°C. The injection lasted 5 min and the needle with the syringe was left in place for 2 min after the injection for the completion of drug infusion. After surgery, mice were housed individually. All experiments were carried out between 9:00 a.m. and 6:00 p.m.

### Plasmids

The overexpression plasmids of GFP, Wnt5a (mouse) and Cyclin D1 (mouse) were incorporated into the pcDNA-Flag expression vector.

### Quantitative Polymerase Chain Reaction (Q-PCR)

Total RNA was extracted and isolated from cells using TRIzol reagent (Invitrogen) as described previously (Peirson and Butler, 2007). First strand cDNA was synthesized from 1 μg of mRNA using Superscript III reverse transcriptase (Invitrogen) and oligo (dT) as primers. Q-PCR was performed in triplicate on an ABI Prism 7000 sequence detection system using an ABI SYBR Green PCR mixture as described by the manufacturer. PCR cycling conditions were as follows: initial denaturation at 95°C for 5–10 min followed by 40 cycles of 95°C for 30 s 1 min of annealing, and 1 min of extension at 72°C. The annealing temperature was adapted for the specific primer set used. Fluorescence data were collected during the annealing stage of amplification and specificity of the amplification was verified by melting curve analysis. Cycle threshold (Ct) values were calculated using identical threshold values for all experiments. *β-actin* was used as a control and for normalization. Relative RNA expression was calculated using the formula ratio = 2 (Ctref−Cttarget). Data shown represent the mean and SE of three separate experiments. The following primer pairs were used: *wnt1* forward (5′-ATG AAC CTT CAC AAC AAC GAG-3′) and reverse (5′-GGT TGC TGC CTC GGT TG-3′); *wnt2* forward (5′-CTG GCT CTG GCT CCC TCT G-3′) and reverse (5′-GGA ACT GGT GTT GGC ACT CTG-3′); *wnt2b* forward (5′-CGT TCG TCT ATG CTA TCT CGT CAG-3′) and reverse (5′-ACA CCG TAA TGG ATG TTG TCA CTA C-3′); *wnt3* forward (5′-CAA GCA CAA CAA TGA AGC AGG C-3′) and reverse (5′-TCG GGA CTC ACG GTG TTT CTC-3′); *wnt3a* forward (5′-CAC CAC CGT CAG CAA CAG CC-3′) and reverse (5′-AGG AGC GTG TCA CTG CGA AAG-3′); *wnt4* forward (5′-GAG AAG TGT GGC TGT GAC CGG-3′) and reverse (5′-ATG TTG TCC GAG CAT CCT GAC C-3′); *wnt5a* forward (5′-CTC CTT CGC CCA GGT TGT TAT AG-3′) and reverse (5′-TGT CTT CGC ACC TTC TCC AAT G-3′); *wnt5b* forward (5′-ATG CCC GAG AGC GTG AGA AG-3′) and reverse (5′-ACA TTT GCA GGC GAC ATC AGC-3′); *wnt6* forward (5′-TGC CCG AGG CGC AAG ACT G-3′) and reverse (5′-ATT GCA AAC ACG AAA GCT GTC TCT C-3′); *wnt7a* forward (5′-ATC TCC GGA TCG GTG ACT TC-3′) and reverse (5′-AGG CCT GGG ATC TTG TTA CAG-3′); *wnt7b* forward (5′-TCT CTG CTT TGG CGT CCT CTA C-3′) and reverse (5′-GCC AGG CCA GGA ATC TTG TTG-3′); *wnt8a* forward (5′-ACG GTG GAA TTG TCC TGA GCA TG-3′) and reverse (5′-GAT GGC AGC AGA GCG GAT GG-3′); *wnt8b* forward (5′-TTG GGA CCG TTG GAA TTG CC-3′) and reverse (5′-AGT CAT CAC AGC CAC AGT TGT C-3′); *wnt9a* forward (5′-GCA GCA AGT TTG TCA AGG AGT TCC-3′) and reverse (5′-GCA GGA GCC AGA CAC ACC ATG-3′); *wnt9b* forward (5′-AAG TAC AGC ACC AAG TTC CTC AGC-3′) and reverse (5′-GAA CAG CAC AGG AGC CTG ACA C-3′); *wnt10a* forward (5′-CCT GTT CTT CCT ACT GCT GCT GG-3′) and reverse (5′-CGA TCT GGA TGC CCT GGA TAG C-3′); *wnt10b* forward (5′-TTC TCT CGG GAT TTC TTG GAT TC-3′) and reverse (5′-TGC ACT TCC GCT TCA GGT TTT C-3′); *wnt11* forward (5′-CTG AAT CAG ACG CAA CAC TGT AAA C-3′) and reverse (5′-CTC TCT CCA GGT CAA GCA GGT AG-3′); *wnt16* forward (5′-AGT AGC GGC ACC AAG GAG AC-3′) and reverse (5′-GAA ACT TTC TGC TGA ACC ACA TGC-3′); *β-actin* forward (5′-CGT CTT CCC CTC CAT CG-3′) and reverse (5′-CTC GTT AAT GTC ACG CAC-3′).

### Immunoblot Analysis

Equal amounts of protein (40–50 mg) were size-fractionated using 6%–15% SDS-PAGE gradient gels. The resolved proteins were electrophoretically transferred onto polyvinylidene difluoride membranes and analyzed by immunoblotting using an ECL chemiluminescence reagent and XAR film (Kodak, XBT-1) according to the manufacturer’s protocol. Primary antibodies were used at optimized dilutions along with the appropriate HRP-conjugated secondary antibodies. The data were collected from at least three independent experiments.

### Immunofluorescence

Brainstem neurons were grown on 13 mm round glass coverslips and processed according to the immunofluorescence protocol as described previously (Xie et al., 2011). Cells were placed on ice, washed twice with PBS and fixed with 0.37% PFA in PBS for 10 min followed by permeabilization with 0.1% Triton X-100 in TBS and blocking in 3% donkey serum. Cells were then washed twice in PBS and treated by cold blocking buffer for 1 h. After sequential treatment with NH_4_Cl (50 mM in 20 mM glycine) for 10 min, the indicated antibody (1:200 in bovine serum albumin) was added and incubated overnight at 4°C. After an additional incubation for 1 h at room temperature with Hoechst 33258 and fluorescein isothiocyanate-conjugated secondary antibody (Invitrogen; 1:400 in bovine serum albumin), the slides were mounted in anti-fading solution (Permafluor, Beckman Coulter, Krefeld, Germany) and stored at 4°C, followed by confocal laser-scanning microscopy. A score of 0–3 was assigned to describe the level of Wnt5a protein based on the red fluorescence area in the cytoplasm of neurons (grade 0, <5%; grade 1, 5%–33%; grade 2, 33%–66%; and grade 3, >66%).

### Subcellular Fractionation

Approximately 10^7^ cells were harvested into 10 ml of isotonic fractionation buffer (250 mM sucrose, 0.5 mM EDTA, 20 mM Hepes, and 500 μM Na_3_VO_4_ at pH 7.2) supplemented with protease inhibitor cocktail complete (Roche Molecular Biochemicals) and centrifuged at 900 *g* for 5 min. The pellet was then resuspended in 200 μl fractionation buffer, homogenized with a ball-bearing homogenizer and centrifuged at 900 *g* for 5 min to enrich the nuclei. The post-nuclear supernatant was centrifuged at 20,000 *g* for 15 min to collect the heavy membrane fraction enriched in mitochondria, the supernatant was as cytoplasm without mitochondrion.

### siRNA Interference

The target sequences for Wnt5a-specific siRNAs were 5′-GAC CUG GUC UAC AUC GAC CTT-3′ and 5′-AGU GCA AUG UCU UCC AAG UTT-3′; Cyclin D1 specific siRNAs were 5′-CCA AUA GGU GUA GGA AAU AGC GCT G-3′ and 5′-AAC ACC AGC TCC TGT GCT GCG-3′ all of which and the negative control siRNA (no silencing small RNA fragment) were synthesized by GenChem Co. (Shanghai, China).

### Cell Survival Assays

Cells were stained with Hoechst 33258 (5 μg/ml) to visualize nuclear morphology. Apoptosis was quantified by scoring the number of cells with pyknotic nuclei (chromatin condensation or nuclear fragmentation) relative to the total number of Hoechst 33258-positive cells in the same visual field. Cells were counted in an unbiased manner (at least 1000 cells for each group) and were scored blindly without previous knowledge of their treatment.

### CaMKII Activity Assay

Homogenates of treated cortical neurons were quantified and used to detect CaMKII activity, using the SignaTECT^®^ Calcium/Calmodulin-Dependent Protein Kinase Assay System (Promega, #V8161) as described by the manufacturer. In brief, the assay quantifies [γ-^32^P]-labeling of a proprietary CaMKII-specific, biotinylated peptide substrate that is based on the T286 autophosphorylation site, followed by streptavidin capture; this recognition sequence is conserved in mouse. Controls include calmodulin and substrate omission. The substrate is specific for CaMKII and does not detect CaMKIV, PKC or calcineurin (Hanson et al., 1989; Goueli et al., 1995). Samples were assayed in triplicate and protein concentration was determined using the bicinchoninic acid (BCA) and Western blotting method. Results were expressed as relative CaMKII activity.

### Tissue Immunohistochemistry

Human cortical tissues obtained from surgical resection of patients with IV grade glioblastoma (glioblastoma adjacent normal cortical tissues) or mouse brains were fixed in 4% paraformaldehyde in 0.1 M phosphate buffer pH 7.6 overnight at 4°C. Dehydration of tissue was through a series of 80%, 95% (v/v) ethanol 1 h each followed by 100% ethanol overnight. Two 100% (v/v) xylene washes were done for 1 h each and then 1 h in 60°C Paraplast Plus (Tyco/Healthcare). After a change of Paraplast Plus, tissue was incubated in a 60°C vacuum oven for 2 h prior to placing in molds to cool and solidify. Sections, 5 μm thick, were cut and mounted. Sections were deparaffinized by drying on superfrost plus slides (Fisher), heating at 56°C overnight, and then washing through mixed xylenes, 100% ethanol, 95% ethanol, ddH_2_O. Slides were immersed in 10 mm citrate buffer, pH 6.0, dry heated for 10 min each to unmask antigen sites, and then cooled and washed in PBS. Endogenous peroxidase activity was inhibited by rinsing the slides in 3% hydrogen peroxide for 5 min. Non-specific binding was blocked by 5 min incubation with the Super Block Solution (ScyTek Laboratories). After washing in PBS, sections were incubated for 30 min at room temperature with indicated antibodies. Sections were washed extensively with PBS and subsequently treated with the Ultra Tek Anti-Polyvalent kit (ScyTek Laboratories). Finally, sections were treated with 3,3′-diaminobenzidine as chromogenic and mounted.

### Statistical Analysis

All experiments were repeated at least three times using independent culture preparations. All measurements were performed blindly. The significance of difference between means was analyzed by the ANOVA and *post hoc* Bonferroni/Dunn tests (for multiple comparisons) and by the Student’s *t* test (for single comparisons). The results are represented as means ± SEM. Statistical significance was determined by value of *p* < 0.05 or *p* < 0.01 for all analyses.

## Results

### Aβ_**42**_ Promotes the Decrease of Wnt5a Expression

**Figure 1:**
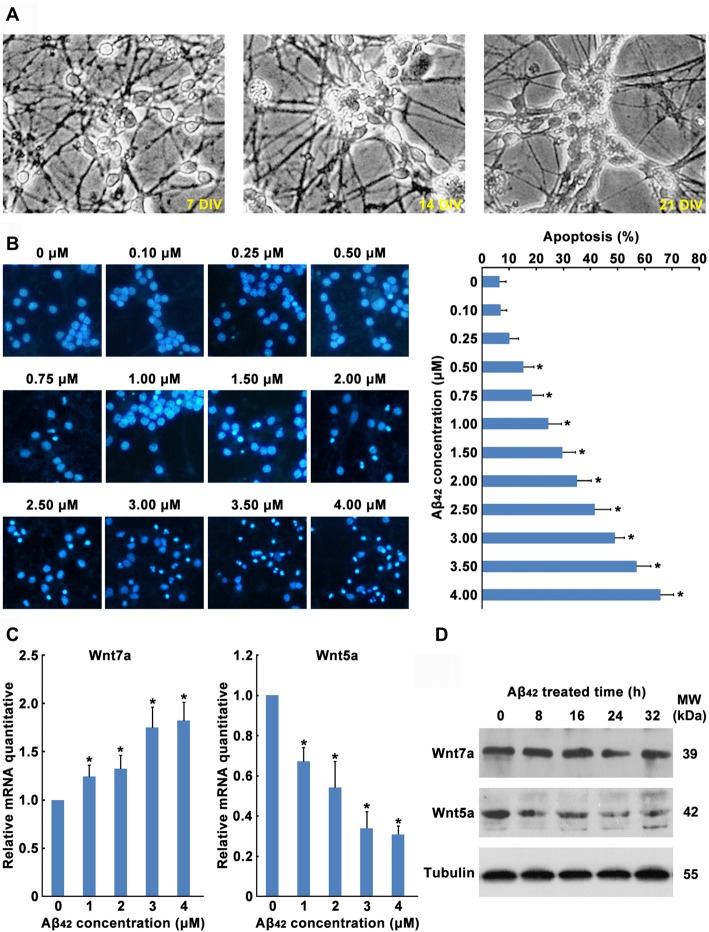
Aβ_42_ promotes the decrease of Wnt5a expression. **(A)** Cortical neurons cultured *in vitro* for 7, 14 and 21 days. **(B)** DIV7 cortical neurons were treated with Aβ_42_ at indicated concentrations for 24 h. Then neurons were stained for nucleus (Hoechst 33258, Blue). Apoptosis was determined by the percentage of cells that were had pyknotic nuclei. **(C)** DIV7 cortical neurons were treated with Aβ_42_ at indicated concentrations for 8 h, total RNA was extracted and analyzed by Q-PCR. **(D)** DIV7 cortical neurons were treated with 3 μM Aβ_42_ at indicated times, total protein was extracted and analyzed by Western blotting. DIV, days *in vitro*; MW, molecular weight; kDa, kilodalton. All data in this figure represent the means ± SEM of three independent experiments. *P < 0.05.

In order to explore the role of Wnt in Aβ_42_ induced neuronal cell death, primary cultured mouse cortical neurons were used in this study (Figure 1A). In the current study, after 5 days culture *in vitro*, cortical neurons were treated with Aβ_42_. We found Aβ caused a concentration-dependent increase of cortical neuron apoptosis (Figure 1B). A significant of cortical neuron apoptosis was observed at concentrations from 0.5 μM to 4 μM (Figure 1B), these results were consistent with previous studies.

Earlier reports have shown that Aβ_42_ may promote neuronal apoptosis by regulating the expression of Wnt family members (Bozyczko-Coyne et al., 2001; Majd et al., 2008). To study which Wnt isoforms take part in accommodation of Aβ_42_ induced cytotoxicity, the mRNA expression levels of all 19 Wnt gene family members in cortical neurons were analyzed by RT-PCR. As illustrated in Table 1, most Wnt members were expressed only at very low levels in both control and Aβ_42_ treated group, Aβ_42_ mainly altered the expression of Wnt5a, Wnt7a, Wnt9b and Wnt11. We next confirmed the mRNA and protein expression levels of these genes. We found the mRNA and protein expression levels of Wnt9b and Wnt11 were undetectable (data not shown); the mRNA expression level of Wnt7a was upregulated after Aβ_42_ treatment, but the protein level of Wnt7a was not changed (Figures 1C,D); Aβ_42_ significantly suppressed the expression level of Wnt5a (Figures 1C,D). These results indicate that Aβ_42_ suppresses the expression of Wnt5a on both mRNA and protein levels.

**Table 1:** Wnts mRNA levels after Aβ_42_ treatment.

Gene name	Control		Aβ_42_ 3 μM for 8 h	
	Fold	CT value	Fold	CT value
Wnt1	1	35.12 ± 0.45	0.85 ± 0.21	32.47 ± 1.54
Wnt2	1	34.51 ± 0.85	1.08 ± 0.19	31.75 ± 1.04
Wnt2b	1	32.17 ± 1.24	0.81 ± 0.28	30.75 ± 0.78
Wnt3	1	33.12 ± 0.67	0.76 ± 0.35	31.45 ± 1.62
Wnt3a	1	35.65 ± 0.58	0.82 ± 0.41	34.07 ± 0.85
Wnt4	1	21.79 ± 0.94	1.07 ± 0.17	21.31 ± 0.84
Wnt5a*	1	24.61 ± 0.52	0.33 ± 0.14	26.35 ± 1.87
Wnt5b	1	31.71 ± 1.62	0.86 ± 0.27	27.32 ± 0.76
Wnt6	1	34.01 ± 0.84	0.91 ± 0.22	31.35 ± 0.94
Wnt7a*	1	36.57 ± 0.59	1.69 ± 0.35	28.52 ± 1.45
Wnt7b	1	30.09 ± 0.77	1.15 ± 0.31	28.82 ± 1.38
Wnt8a	1	34.21 ± 1.46	0.79 ± 0.47	35.58 ± 0.65
Wnt8b	1	32.83 ± 1.56	0.82 ± 0.02	36.14 ± 0.53
Wnt9a	1	28.94 ± 1.84	1.12 ± 0.35	29.25 ± 0.84
Wnt9b*	1	35.08 ± 1.41	2.63 ± 0.42	35.55 ± 0.67
Wnt10a	1	34.51 ± 1.05	1.24 ± 0.39	33.95 ± 0.72
Wnt10b	1	33.75 ± 0.62	0.87 ± 0.23	33.47 ± 1.07
Wnt11*	1	33.44 ± 1.21	2.14 ± 1.39	36.54 ± 0.81
Wnt16	1	37.25 ± 1.44	0.91 ± 0.27	31.28 ± 0.65

The values represent the mean ± SE of three independent experiments. *P < 0.05.

### Aβ_42_ Inhibited the Expression of Wnt5a *in Vivo*

**Figure 2:**
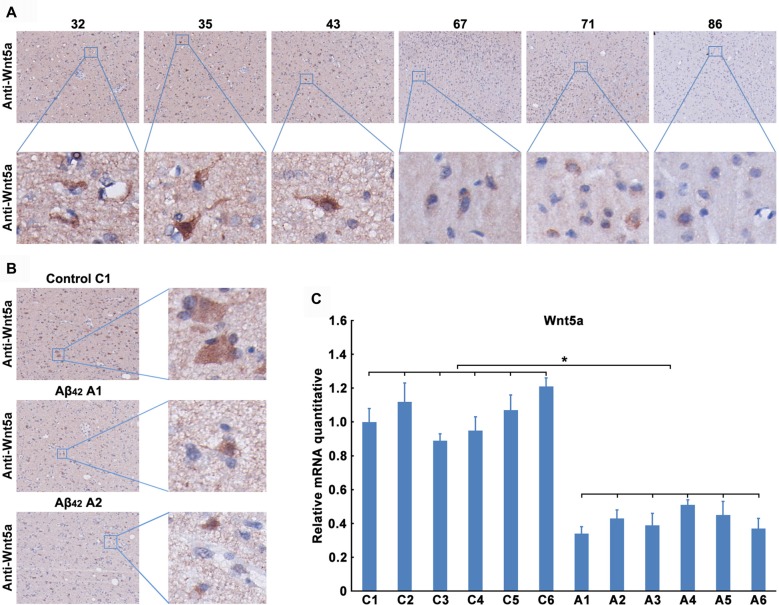
Aβ_42_ inhibited the expression of Wnt5a *in vivo*. **(A)** Wnt5a immunoreactivity was primarily detected in the adjacent normal brain tissues of malignant glioma from 32, 35, 43, 67, 71 and 86 years old patients (magnification of upper panel: ×40; magnification of lower panel: ×400). **(B,C)** A solution of Aβ_42_ (3 nmol/10 μl) or Aβ_42_ (3 nmol/10 μl) combined with recombinant Wnt5a protein (6 nmol/10 μl) was injected into the lateral ventricles of mice. Seven days after treatment, Wnt5a immunoreactivity was primarily detected in mouse brain tissues (**B**, control C1: control group mouse 1; Aβ_42_ A1: Aβ_42_ treated group mouse 1; Aβ_42_ A2: Aβ_42_ treated group mouse 2; magnification of left panel: ×40; magnification of right panel: ×400), total RNA was extracted and analyzed by Q-PCR (**C**, C1–C6: control group mouse 1–6; A1–A6: Aβ_42_ treated group mouse 1–6). All data in this figure represent the means ± SEM of three independent experiments. *P < 0.05.

We next determined the expression level of Wnt5a protein in human brains. Unfortunately, we were unable to obtain postmortem brains derived from AD patients. As an alternative, we collected the adjacent normal brain tissues of malignant glioma from 32- to 86-year-old patients. Immunohistochemical staining of the adjacent normal brain tissues of malignant glioma revealed that Wnt5a levels were consistently expressed in the neurons, this result was consistent with previous study (Howng et al., 2002); however, the Wnt5a levels were decreased with the increase of age (Figure 2A). These results indicate a role of Wnt5a in the aging of human brain.

To examine the effect of Aβ_42_ on the expression of Wnt5a *in vivo*, a solution of Aβ_42_ was injected into the lateral ventricles of mice. Seven days after the injection, tissues from the cerebral cortex were collected to assess the expression of Wnt5a. We found that the protein level of Wnt5a was decreased in cortical neurons after Aβ_42_-treatment (Figure 2B). This observation was further supported by quantitative PCR analysis (Figure 2C). Therefore, Aβ_42_ suppresses the expression of Wnt5a on both mRNA and protein levels *in vivo*.

### Wnt5a Downregulation Contributes to Aβ_**42**_ Induced Cortical Neuron Apoptosis

**Figure 3:**
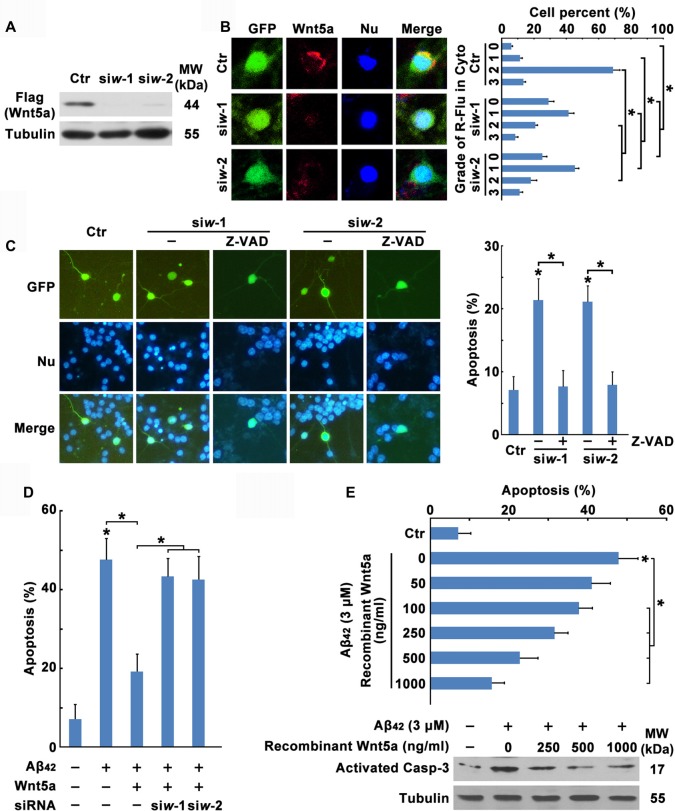
Wnt5a downregulation contributes to Aβ_42_ induced cortical neuron apoptosis. **(A)** HEK239 cells were transfected with Flag-Wnt5a and Wnt5a siRNAs for 12 h, then cells were subjected to immunoblot detection. **(B)** DIV6 cortical neurons were transfected with GFP plasmids and Wnt5a siRNAs for 24 h. Immunofluorescence detection of Wnt5a of neurons appearing in Red, then neurons were stained for nucleus (Blue; left panel). The efficiency of Wnt5a siRNAs were estimated by a score of 0–3 based on the red fluorescence area in the cytoplasm of neurons (grade 0, <5%; grade 1, 5%–33%; grade 2, 33%–66%; and grade 3, >66%; right panel). **(C)** DIV6 cortical neurons were transfected with GFP plasmids and Wnt5a siRNAs for 12 h following 50 μM Z-VAD treatment for another 12 h, and then neurons were stained for nucleus (Blue). Apoptosis was determined by the percentage of GFP positive cells that were had pyknotic nuclei. **(D)** DIV6 cortical neurons were transfected with GFP plasmids, Wnt5a plasmids and Wnt5a siRNAs for 24 h, and then neurons were stained for nucleus (Blue). Apoptosis was determined by the percentage of GFP positive cells that were had pyknotic nuclei. **(E)** DIV6 cortical neurons were treated with 3 μM Aβ_42_ with or without recombinant Wnt5a protein at indicated concentrations for 24 h, and then neurons were stained for nucleus (Blue). Apoptosis was determined by the percentage of cells that were had pyknotic nuclei (upper panel); or cells were subjected to immunoblot detection (lower panel). MW, molecular weight; kDa, kilodalton. All data in this figure represent the means ± SEM of three independent experiments. *P < 0.05.

To investigate the function of Wnt5a in cortical neuron survival and apoptosis, two siRNAs were designed to reduce mouse Wnt5a protein levels. These siRNAs completely abolished the expression of a transfected mouse Flag-Wnt5a construct in HEK293 cells (Figure 3A). In cortical neurons, Wnt5a siRNAs were co-transfected with the marker plasmid pCMV-GFP, immunofluorescence analysis using anti-Wnt5a antibody revealed that the expression of Wnt5a was abolished by the two Wnt5a siRNAs (Figure 3B), demonstrating the efficacy of the siRNAs in cortical neurons. Furthermore, siRNAs specifically targeted against Wnt5a induced cortical neuron apoptosis, and the Wnt5a siRNAs induced apoptosis was inhibited by Caspase inhibitor Z-VAD (Figure 3C). These results indicate that Wnt5a is required for the survival of cortical neuron.

To further confirm the effect of Wnt5a on cortical neuron survival, transfection of neurons with wild-type Wnt5a also significantly increased neuron survival during Aβ_42_ treatment, and the effect induced by Wnt5a overexpression was abolished by Wnt5a siRNAs (Figure 3D). Moreover, we added various concentration (0, 50, 100, 250, 500, 1000 ng/ml) of recombinant Wnt5a protein to the culture medium of Aβ_42_ treated neurons. The suppressive effect of recombinant Wnt5a protein on Aβ_42_ induced neuronal apoptosis was dose-dependent (Figure 3E). Taken together, Wnt5a acts as a mediator of cortical neuron survival, and Aβ_42_ promotes cortical neuron apoptosis by downregulating the expression of Wnt5a.

### Wnt5a Inhibits the Expression of Cyclin D1

Neurons in culture exposed to Aβ_42_ could re-enter the cell cycle, cross the G1-S-phase transition and begin *de novo* DNA synthesis before apoptotic death occurs (Caricasole et al., 2003; Majd et al., 2008). And cell-cycle activation is causally related to neuronal death (Busser et al., 1998; Giovanni et al., 1999; Liu and Greene, 2001; Yang et al., 2003; Becker and Bonni, 2004). Meanwhile, Wnt pathway is the key pathway in activation of cell cycle (Davidson and Niehrs, 2010). Interestingly, Wnt5a can act as a cell proliferation inhibitor in many cancer cells and mouse B cells (Liang et al., 2003; Kremenevskaja et al., 2005; Bitler et al., 2011), suggesting a cell-cycle suppressor role for Wnt5a in some cell types. We speculate that Aβ may activate the cell-cycle pathway by reducing the expression of Wnt5a.

**Figure 4:**
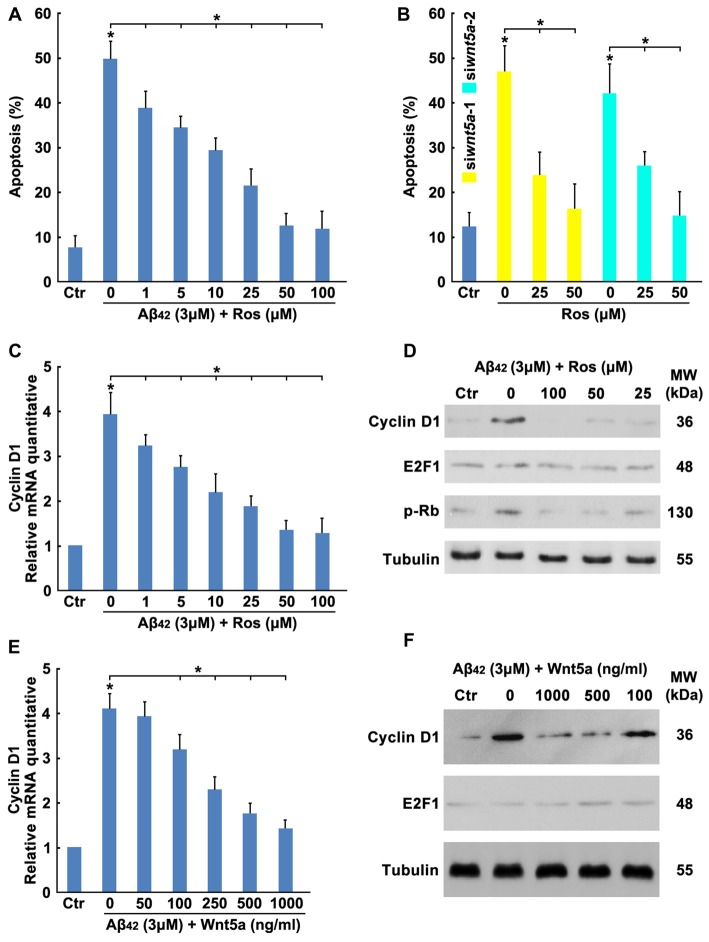
Wnt5a inhibits the expression of Cyclin D1. **(A)** DIV6 cortical neurons were treated with 3 μM Aβ_42_ with or without Roscovitine (ROS) at indicated concentrations for 24 h, then apoptosis was determined. **(B)** DIV6 cortical neurons were treated with Wnt5a siRNAs and ROS at indicated concentrations for 24 h, then apoptosis was determined. **(C)** DIV6 cortical neurons were treated with 3 μM Aβ_42_ with or without ROS at indicated concentrations for 12 h, total RNA was extracted and analyzed by Q-PCR. **(D)** DIV6 cortical neurons were treated with 3 μM Aβ_42_ with or without ROS at indicated concentrations for 16 h, total protein was extracted and analyzed by Western blotting. **(E)** DIV6 cortical neurons were treated with 3 μM Aβ_42_ with or without recombinant Wnt5a protein at indicated concentrations for 12 h, total RNA was extracted and analyzed by Q-PCR. **(F)** DIV6 cortical neurons were treated with 3 μM Aβ_42_ with or without recombinant Wnt5a protein at indicated concentrations for 16 h, total protein was extracted and analyzed by Western blotting. MW, molecular weight; kDa, kilodalton. All data in this figure represent the means ± SEM of three independent experiments. *P < 0.05.

To determine the effect of Aβ_42_ in inducing cortical neuron to re-enter the cell cycle, neurons were treated with Aβ_42_ combined with or without CDKs inhibitor ROS (Bach et al., 2005), results showed that Aβ_42_ induced neuronal apoptosis was significantly decreased by CDKs inhibitor (Figure 4A). Furthermore, CDKs inhibitor also inhibited Wnt5a siRNAs induced cortical neuron apoptosis (Figure 4B). These findings demonstrate that Aβ_42_ could promote neurons re-enter the cell cycle by reducing the expression of Wnt5a.

Aberrations in cell cycle control are always controlled by cell cycle regulators, such as cyclins, Myc and E2F1 (van den Heuvel, 2005). To study which cell cycle regulators mediated the Aβ_42_ activated cell cycle, the mRNA expression levels of cyclins, Myc and E2F1 in cortical neurons were analyzed by RT-PCR. As illustrated in Table 2, most cyclin members and Myc were expressed only at very low levels in both control and treatment group, Aβ_42_ mainly altered the expression of Cyclin D1 and E2F1. We next confirmed that Aβ_42_ significantly increased the expression level of Cyclin D1, and the upregulation of Cyclin D1 was eliminated by CDKs inhibitor ROS (inhibited the CDK mediated phosphorylation of Rb Ser795; Figures 4C,D). Accordingly, neurons treated with recombinant Wnt5a protein also abolished Aβ_42_ induced Cyclin D1 expression (Figures 4E,F). However, the protein level of E2F1 was not changed after Aβ_42_, CDKs inhibitor and Wnt5a treatment (Figures 4C,D). Taken together, these results suggest that Wnt5a prevents cell cycle activation by inhibiting the expression of Cyclin D1 in cortical neurons.

**Table 2:** Cell cycle regulators mRNA levels after Aβ_42_ treatment.

Gene name	Control		Aβ_42_ 3 μM for 12 h	
	Fold	CT value	Fold	CT value
Cyclin A1	1	28.34 ± 1.21	1.04 ± 0.27	29.71 ± 1.45
Cyclin A2	1	29.44 ± 0.85	0.93 ± 0.21	30.11 ± 2.18
Cyclin B1	1	25.79 ± 1.57	1.12 ± 0.27	26.89 ± 1.37
Cyclin B2*	1	36.51 ± 2.44	1.67 ± 0.35	35.77 ± 1.86
Cyclin B3	1	35.74 ± 2.58	2.81 ± 1.52	37.94 ± 1.81
Cyclin D1*	1	24.27 ± 0.41	3.72 ± 0.38	23.17 ± 0.92
Cyclin D2	1	29.13 ± 1.26	1.24 ± 0.35	28.77 ± 1.81
Cyclin D3*	1	32.11 ± 1.89	1.51 ± 0.47	33.24 ± 0.53
Cyclin E1	1	36.17 ± 2.31	2.14 ± 1.22	37.18 ± 0.72
Cyclin E2*	1	35.51 ± 2.64	2.05 ± 0.41	36.19 ± 2.48
Myc*	1	38.63 ± 1.75	2.15 ± 0.89	39.04 ± 1.51
E2F1*	1	27.89 ± 0.31	2.57 ± 0.63	26.55 ± 0.76

The values represent the mean ± SE of three independent experiments. *P < 0.05.

### Wnt5a Inhibits the Expression of Cyclin D1 *in Vivo*

**Figure 5:**
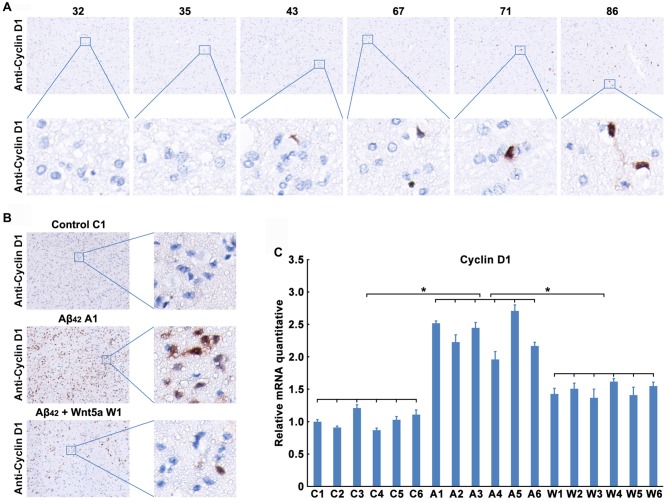
Wnt5a inhibits the expression of Cyclin D1 *in vivo*. **(A)** Cyclin D1 immunoreactivity was primarily detected in the adjacent normal brain tissues of malignant glioma from 32, 35, 43, 67, 71 and 86 years old patients (magnification of upper panel: ×40; magnification of lower panel: ×400). **(B,C)** A solution of Aβ_42_ (3 nmol/10 μl) or Aβ_42_ (3 nmol/10 μl) combined with recombinant Wnt5a protein (6 nmol/10 μl) was injected into the lateral ventricles of mice. Seven days after treatment, Cyclin D1 immunoreactivity was primarily detected in mouse brain tissues (**B**, control C1: control group mouse 1; Aβ_42_ A1: Aβ_42_ treated group mouse 1; Aβ_42_ + Wnt5a W1: Aβ_42_ and Wnt5a treated group mouse 1; magnification of left panel: ×40; magnification of right panel: ×400), total RNA was extracted and analyzed by Q-PCR (**C**, C1–C6: control group mouse 1–6; A1–A6: Aβ_42_ treated group mouse 1–6; W1–W6: Aβ_42_ and Wnt5a treated group mouse 1–6). All data in this figure represent the means ± SEM of three independent experiments. *P < 0.05.

We next confirmed the expression level of Cyclin D1 protein in the adjacent normal brain tissues of malignant glioma from 32- to 86-year-old patients. As observed in previous studies (Hoozemans et al., 2002), trace amounts of Cyclin D1 was detected in the brain cortex of 32, 35 and 43 years old people, but the protein levels were upregulated with the increase of age (Figure 5A). These results also indicated a relationship between the neuronal aging and Cyclin D1.

To examine the effect of Wnt5a on Cyclin D1 *in vivo*, a solution of Aβ_42_ or Aβ_42_ combined with recombinant Wnt5a protein was injected into the lateral ventricles of mice. Seven days after the injection, tissues from the cerebral cortex were collected to assess the expression of Cyclin D1. We found that the protein level of Cyclin D1 was increased in cortical neurons after Aβ_42_-treatment, and the increase of Cyclin D1 was inhibited by recombinant Wnt5a protein (Figure 5B). This observation was further supported by quantitative PCR analysis (Figure 5C). These *in vivo* results support the conclusion that Wnt5a could prevent Aβ_42_ induced Cyclin D1 expression.

### Wnt5a/CaMKII Pathway Down-Regulates the Expression of Cyclin D1

**Figure 6:**
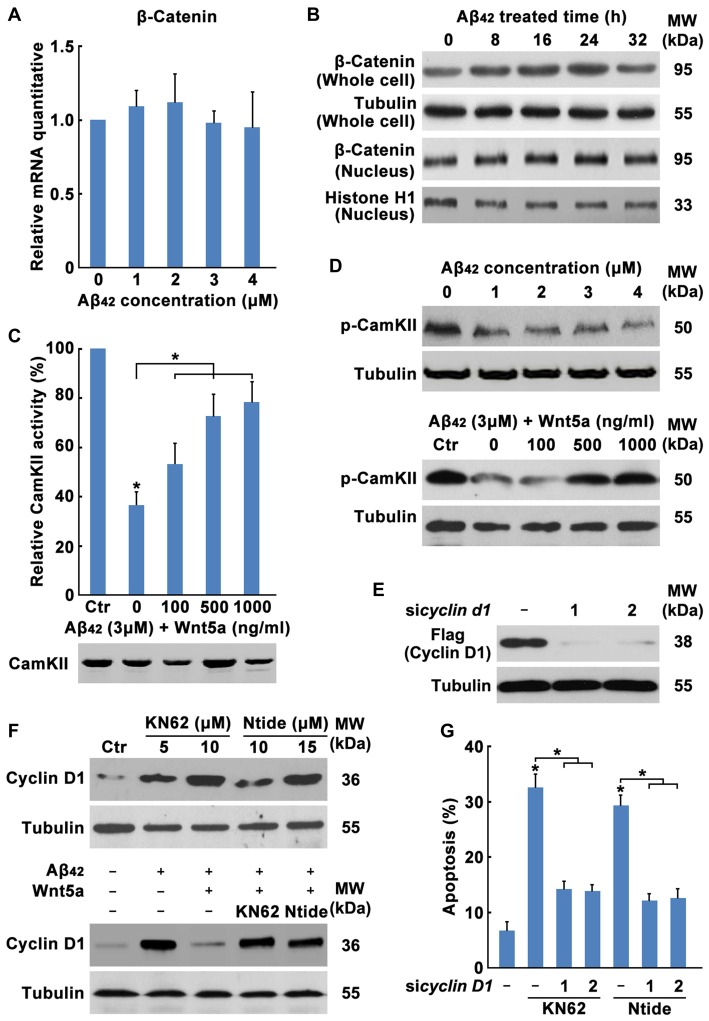
Wnt5a/CaMKII pathway down-regulates the expression of Cyclin D1. **(A)** DIV7 cortical neurons were treated with Aβ_42_ at indicated concentrations for 12 h, total RNA was extracted and analyzed by Q-PCR. **(B)** DIV7 cortical neurons were treated with 3 μM Aβ_42_ at indicated times, total protein or nuclear protein was extracted and analyzed by Western blotting. **(C)** DIV6 cortical neurons were treated with 3 μM Aβ_42_ with or without recombinant Wnt5a protein at indicated concentrations for 12 h. CamKII activity was determined using SignaTECT^®^ Calcium/Calmodulin-Dependent Protein Kinase Assay System (Upper panel). The input total CamKII was analyzed by Western blotting (Lower panel). **(D)** DIV7 cortical neurons were treated with Aβ_42_ at indicated concentrations (Upper panel) or treated with 3 μM Aβ_42_ with/without recombinant Wnt5a protein at indicated concentrations (Lower panel) for 16 h, total protein was extracted and analyzed by Western blotting. **(E)** DIV7 cortical neurons were treated with KN62 or Calmodulin Kinase II Ntide, Myristoylated (Ntide) at indicated concentrations for 16 h (Left panel), or DIV7 cortical neurons were treated with 3 μM Aβ_42_ with/without 500 ng/ml recombinant Wnt5a protein or 10 μM KN62 or 15 μM Ntide as indicated for 16 h (Right panel), total protein was extracted and analyzed by Western blotting. **(F)** HEK239 cells were transfected with Flag-Cyclin D1 and Cyclin D1 siRNAs for 12 h, then cells were subjected to immunoblot detection. **(G)** DIV7 cortical neurons were treated with 10 μM KN62 or 15 μM Ntide and transfected with or without Cyclin D1 siRNAs for 24 h (Left panel), then apoptosis was determined. MW, molecular weight; kDa, kilodalton. All data in this figure represent the means ± SEM of three independent experiments. *P < 0.05.

The mechanism of Wnt5a-mediated inhibition of the Cyclin D1expression is unclear. Wnt5a has been proposed to activate both the canonical Wnt/β-catenin pathway (He et al., 1997; Toyofuku et al., 2000) and Wnt/Ca^2+^ pathway (Liang et al., 2003). Activation of the Wnt/Ca^2+^ pathway has been suggested to block the Wnt/β-catenin signaling cascade (Torres et al., 1996; Kühl et al., 2001; Ishitani et al., 2003). To dissect the mechanistic pathway utilized by Wnt5a in regulating cortical neuron cell-cycle activation, we analyzed β-catenin levels in Aβ_42_ treated and untreated neurons. No difference was detected in β-catenin levels in whole-cell or in nuclear extracts between Aβ_42_ treated and untreated neurons, suggesting that Wnt5a was neither activating nor inhibiting the canonical Wnt/β-catenin pathway in cortical neuron (Figures 6A,B).

Therefore, we examined the Wnt/Ca^2+^ pathway. CamKII activity assays showed that Aβ_42_ treated cells possess 63.51% less activity than Aβ_42_ untreated cells and the decrease of CamKII activity was recovered by Wnt5a recombinant protein stimulation (Figure 6C), even though Western blotting analysis revealed equal amounts of total CamKII in both samples (Figure 6C). Furthermore, we examined the level of phospho-CaMKII alpha (Thr286)/beta (Thr287) by Western blotting. CaMKII phosphorylation was decreased at as early as 0.5 h after Aβ_42_ treatment and was also recovered by Wnt5a recombinant protein stimulation (Figure 6D). These results indicate that Wnt5a is signaling via the Wnt/Ca^2+^ pathway to activate CamKII.

To further confirm the ability of Wnt/Ca^2+^/CaMKII pathway to downregulate Cyclin D1 and inhibit cell-cycle activation, KN62 [a pan-inhibitor of CaMKs (Tokumitsu et al., 1990)] and CaMKII-Ntide [an endogenous selective inhibitor of CaMKII (Chang et al., 1998)], was used to treat cortical neurons. Western blotting analysis revealed that these two inhibitors elevated Cyclin D1 expression of both Aβ_42_ untreated and Aβ_42_ plus Wnt5a recombinant protein treated neurons (Figure 6E). Furthermore, we found CamKII inhibitors were sufficient to cause cortical neuron apoptosis in a Cyclin D1 dependent way (Figures 6F,G). Collectively, Wnt5a/CaMKII pathway down-regulates the expression of Cyclin D1.

### Wnt5a Promotes Cortical Neuron Survival by Inhibiting Cyclin D1 Expression

**Figure 7:**
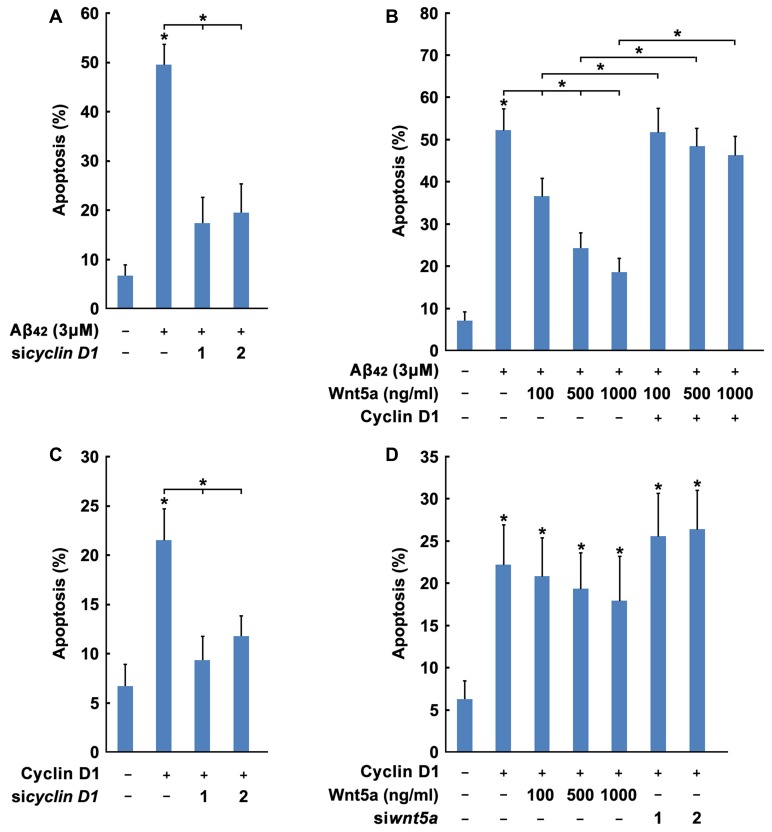
Wnt5a promotes cortical neuron survival by inhibiting Cyclin D1 expression. **(A)** DIV6 cortical neurons were transfected with GFP plasmids and Cyclin D1 siRNAs for 12 h, and treated with 3 μM Aβ_42_ for another 12 h. Apoptosis was determined by the percentage of GFP positive cells that were had pyknotic nuclei. **(B)** DIV6 cortical neurons were transfected with GFP plasmids, Flag-Cyclin D1 plasmids and Cyclin D1 siRNAs for 24 h. Apoptosis was determined by the percentage of GFP positive cells that were had pyknotic nuclei. **(C)** DIV6 cortical neurons were transfected with or without GFP plasmids, Flag-Cyclin D1 plasmids or control plasmids for 12 h, then treated with 3 μM Aβ_42_ with/without recombinant Wnt5a protein at indicated concentrations for 24 h. Apoptosis was determined by the percentage of GFP positive cells that were had pyknotic nuclei. **(D)** DIV6 cortical neurons were transfected with or without GFP plasmids, Flag-Cyclin D1 plasmids or Cyclin D1 siRNAs for 12 h, then treated with with/without recombinant Wnt5a protein at indicated concentrations for 24 h. Apoptosis was determined by the percentage of GFP positive cells that were had pyknotic nuclei. MW, molecular weight; kDa, kilodalton. All data in this figure represent the means ± SEM of three independent experiments. *P < 0.05.

Previous studies have established that overexpression of Cyclin D1 may lead to neuronal apoptosis (Kranenburg et al., 1996). To assess whether Cyclin Dl overexpression would induce this phenomenon, two siRNAs were designed to reduce mouse Cyclin D1 protein levels (Figure 6F). siRNAs specifically targeted against Cyclin D1 inhibited Aβ_42_ induced cortical neuron apoptosis (Figure 7A). Furthermore, we confirmed that overexpression of Cyclin D1 could lead to cortical neuron apoptosis, and Cyclin D1 induced apoptosis was also abolished by Cyclin D1 siRNAs (Figure 7B). These results indicate that Cyclin D1 is required for Aβ_42_ induced cortical neuron apoptosis.

Based on the obvious importance of Wnt5a and Cyclin D1 in cortical neuron survival and apoptosis, we hypothesized that Wnt5a might mediate the cortical neuron survival by inhibiting Cyclin D1 expression. Manipulations of Wnt5a and Cyclin D1, using knockdown, overexpression or recombinant protein approaches, were combined to investigate their functional relationship. The Wnt5a recombinant protein significantly blocked cortical neuron apoptosis in Aβ media (Figure 7C). In contrast, overexpression of wild-type Cyclin D1 removed the protection conferred by Wnt5a recombinant protein (Figure 7C). Overexpression of wild-type Cyclin D1 induced apoptosis could not prevent by adding Wnt5a recombinant protein, and the knockdown of endogenous Wnt5a also could not enhance the apoptosis rate (Figure 7D). These findings demonstrated that Cyclin D1 lies downstream of the Wnt5a-dependent pro-survival signaling cascade.

## Discussion

Wnt signaling has a key role in the nervous system (Salinas, 2012; Inestrosa and Varela-Nallar, 2014; Lambert et al., 2016). On the one hand, Wnt signaling plays critical roles in several physiological cellular processes of neurons, including regulate the differentiation and migration of neural progenitor cells, participate in the formation of neuronal circuits, playing roles in dendrite and axon development, dendritic spine formation (Inestrosa and Arenas, 2010; Budnik and Salinas, 2011; Salinas, 2012). On the other hand, the Wnt pathway modulate the survival of mature neurons and aberrant signaling by Wnt pathways is linked to a range of neurodegenerative diseases (Inestrosa and Varela-Nallar, 2014; Purro et al., 2014; Libro et al., 2016; Zhang et al., 2016). Interestingly, previous studies have reported that stimulation of Wnt signaling by exogenous Wnts could protect cultured neurons against Aβ-mediated neurotoxicity (Cerpa et al., 2010; Silva-Alvarez et al., 2013). Exogenous Wnt5a was able to modulate the increase in the anti-apoptotic protein on the outer mitochondrial membrane of hippocampal neurons (Cerpa et al., 2010), suggesting that Wnt5a may protects neurons from the apoptotic effect induced by Aβ oligomers. Wnt5a also plays a pivotal role in the maintenance of normal postsynaptic integrity (Cerpa et al., 2010), and its activation may be of therapeutic interest in patients with AD. However, there is only one factor Dickkopf 1 (Dkk1), a neurodegenerative factor that serves as an antagonist of the canonical Wnt signaling pathway, has been report to inhibit Wnt activity and induct of neuronal cell death (Caricasole et al., 2004; Scali et al., 2006), the direct role of endogenous Wnt family members play in neurodegenerative diseases is still unknown. For the first time, we report that Wnt5a signals through the noncanonical Wnt/Ca^2+^ pathway to suppress cyclin D1 expression and negatively regulate neuronal cell death by inhibitiing cell-cycle activation.

Cell-cycle activation is causally related to Aβ induced neuronal death (Caricasole et al., 2003; Varvel et al., 2008). In this study, we find endogenous Wnt5a increase the survival of cortical neurons by inhibiting the cell-cycle activation and cell proliferation, and the downregulation of Wnt5a contributes to Aβ toxicity in neuron cultures. Many studies have also reported that administration of exogenous Wnt5a prevented Aβ-induced synaptotoxicity and promoted neuronal survival (Varvel et al., 2008; Varela-Nallar et al., 2012; Silva-Alvarez et al., 2013; Godoy et al., 2014). Recently, Wnt5a has been showed to up-regulated in mouse brains prior to AD phenotypes and in Aβ treated cortical neurons (Li et al., 2011). We think the difference between our and previous studies (Li et al., 2011) may induced by different experimental method. We used specific siRNAs to knockdown the expression of Wnt5a; but Li et al. (2011) used an rabbit-anti-Wnt5a antibody (from Abcam) to perform immunohistochemistry experiment and detect endogenous AD mouse brain Wnt5a, they also used this antibody to suppress Wnt5a signaling. Interestingly, we have tested the same antibody by Western blotting, we found this antibody coud interact with many nonspecific proteins (data not shown), and this Wnt5a antibody may induce non-specific results during the experiment. In fact, Wnt5a has been shown to play different roles in the regulation of neuronal cell-cycle activation and following cell proliferation during different neural cell development stage (Inestrosa and Arenas, 2010). Wnt5a can promote the proliferation of cultured neuronal progenitor cells isolated from postnatal and adult mouse SvZ (subventricular zone of the lateral ventricles in the forebrain), but reduce dopaminergic progenitor proliferation and neurogenesis to promote ventral midbrain morphogenesis in loss-of-function experiments *in vivo* (Inestrosa and Arenas, 2010), indicating Wnt5a may play as an inhibitor of cell-cycle activation in mature neurons.

Development studies and cell differentiation studies using various model systems have revealed that Wnt5a can signal through both Wnt/β-catenin pathway and Wnt/Ca^2+^ pathway to regulate cell adhesion, mobility, proliferation, and differentiation (He et al., 1997; Sheldahl et al., 1999; Toyofuku et al., 2000; Kühl et al., 2001). To determine which pathway mediates Wnt5a signaling in mature cortical neurons, we first found expression of Myc or E2F1 was unchanged, expression of cyclin D1 was decreased after Aβ treatment. Whole-cell and nuclear β-catenin level remained unchanged in the presence or absence of Aβ, while activity of CamKII was decreased in Wnt5a null or Aβ treated cells. These results indicate that Wnt5a signals via the Wnt/Ca^2+^ pathway to inhibit cyclin D1 expression. Because β-catenin levels and Myc expression levels were unchanged in the presence or absence of Wnt5a, it is unlikely that Wnt5a inhibits the Wnt/β-catenin signaling cascade in these cells. However, the mechanism that how Wnt5a signaling activates the Ca^2+^ pathway to inhibit cyclin D1 expression need further study.

In this study, we encountered some confusing phenomenon. Due to the low transfection efficiency of cortical neurons (<1%), we have to validate the effectiveness of siRNA in HEK293 cells (transfection efficiency >95%). We show that siRNAs against Wnt5a results in a complete loss of expression in HEK293 cells. However, these Wnt5a siRNAs only induces apoptosis in about 20% of transfected cortical neurons. We think there are two reasons causing this result: (1) in HEK293 cells, Wnt5a siRNAs and Flag-Wnt5a expression plasmids were transfected into cells at the same time, the expression of exogenous Flag-Wnt5a was inhibited at the very beginning. Therefore, a complete inhibitory effect has seen in HEK293 cells; and (2) in neurons, both the mRNA and protein levels of endogenous Wnt5a are high. Thus, the Wnt5a siRNAs can only partially reduce the level of endogenous Wnt5a in the transfected neurons. To evaluate the exact effect of Wnt5a siRNAs, a score of 0–3 was assigned to describe the levels of Wnt5a protein based on the red fluorescence area in the cytoplasm of neurons (grade 0, <5%; grade 1, 5%–33%; grade 2, 33%–66%; and grade 3, >66%; Figue ue 3B). After Wnt5a siRNAs treatment, the ratio of cortical neurons in grade 0 increased from 6% to 25%–30%, and this is consistent with the percent of cortiacl neuron apoptosis induced by Cyclin D1 siRNAs.

## Conclusion

Our studies reveal that Wnt5a increases neuronal survival by negative regulating cell-cycle activation *in vitro* and *in vivo*. This conclusion is supported in part by previous experiments in which adding exogenous Wnt5a in hippocampal neurons and cortical neurons. The downregulation of Wnt5a signaling might be an important contributor of AD-related neurodegeneration.

## Author Contributions

LZ conceived of the study, and participated in its design and helped to draft the manuscript; LZ also carried out the cell culture, Q-PCR, pathological studies, animal surgery, immunoblot analysis, CaMKII activity assay and cell survival assays. DC and Y-GW carried out the cell culture, animal surgery and plasmid construct. X-MH and FL carried out the Q-PCR, animal surgery and immunofluorescence. HC, ST, Y-LS and WC carried out the subcellular fractionation. W-XY carried out the tissue immunohistochemistry. Z-CC carried out the siRNA interference. Z-JL, Z-ZD and WC carried out the immunoblot analysis. R-HY carried out the pathological studies and tissue immunohistochemistry. TP, NJ and M-FH conceived of the study and participated in its design. Z-QL conceived of the study, and participated in its design and coordination and helped to draft the manuscript; Z-QL also carried out the pathological studies and animal surgery. All authors read and approved the final manuscript.

## Conflict of Interest Statement

The authors declare that the research was conducted in the absence of any commercial or financial relationships that could be construed as a potential conflict of interest.
